# Chronic Hepatitis C: Pathophysiology and Clinical Issues

**DOI:** 10.3390/biology11121737

**Published:** 2022-11-29

**Authors:** Maria Lorena Abate, Gian Paolo Caviglia

**Affiliations:** Department of Medical Sciences, University of Turin, 10126 Turin, Italy

Globally, it is estimated that 56.8 million people are chronically infected with hepatitis C virus (HCV) [1]. The natural history of chronic hepatitis C (CHC) is characterized by a persistent liver inflammation that progressively leads to fibrosis accumulation and eventually to cirrhosis [2], which is the principal risk factor for the development of hepatocellular carcinoma (HCC) [3,4]. 

Until 2011, the standard of care for CHC was dual therapy based on pegylated interferon (Peg-IFN) in combination with ribavirin (RBV) [5], resulting in rates of sustained virologic response (SVR) ranging from 40% to 80% according to HCV genotypes [6,7]. Thereafter, the approval of the first generation of protease inhibitors, namely telaprevir and boceprevir, led to remarkable results, doubling the cure rate for patients chronically infected with HCV genotype 1 [8]. However, the second generation of antiviral agents with direct activity against HCV represented the real revolution for the treatment of CHC, sofosbuvir (SOF) being the herald of a new class of oral direct-acting antiviral (DAA) agents; these drugs achieved very high SVR rates (90–100%), improved tolerability, and reduced treatment duration [9].

Despite the remarkable improvements in the therapeutic setting, different biological and clinical aspects related to chronic HCV infection deserve further investigation. This Special Issue provides further knowledge on the residual risk of liver cirrhosis complications in patients successfully treated with DAAs, the potential strategies to expand the screening and treatment of special populations and at-risk groups, the impact of non-hepatotropic viruses on liver disease severity, and the extrahepatic manifestations of CHC.

Armandi and colleagues showed that HCV eradication by DAA has a significant impact on the natural history of CHC, even in patients with cirrhosis [10]: among 373 patients with HCV-related cirrhosis treated with DAA, the authors observed a higher proportion of patients with improved liver function as well as liver stiffness (19.3 kPa vs. 11.6 kPa, *p* < 0.001) after 6 months of follow-up (FU) [10]. However, patients with cirrhosis are still at risk of HCC development, even if their HCV is cure [11]. Here, we reported that the measurement of serum epidermal growth factor receptor 3 in HCV patients with a diagnosis of HCC was able to predict overall survival irrespectively from patients’ age, liver function and tumor stage [12]. 

Another relevant issue is the implementation of a micro-elimination approach in order to achieve the global elimination of HCV by dedicated programs for well-defined population segments [13]. In this regard, Nevola and colleagues provided an in-depth review of the role and impact of telemedicine for the optimization of care in difficult-to-treat patients [14]. Furthermore, the same group showed the results of an innovative HCV treatment approach, employing a telemedicine-based hepatological stewardship for people who use drugs [15]. The authors enrolled 135 patients with active HCV infection; all patients started treatment within 3 weeks from HCV diagnosis. Remarkably, only six drop-outs were recorded, while 98.5% of patients obtained an SVR at week 12 [15].

Yurlov and colleagues focused their attention on non-hepatotropic viruses as a cause or contributing cause in the occurrence, progression, and outcome of inflammatory liver diseases [16]. Specifically, the authors observed a high prevalence of cytomegalovirus, Epstein–Barr virus, and human herpesviruses (HHV) type 6 in biological samples of patients with liver cirrhosis as compared to those without advanced liver disease; furthermore, patients with herpesvirus coinfection showed a trend toward high rates of liver decompensation, hepatorenal syndrome, and portal hypertension [16]. Finally, Priora and colleagues recapitulated the rheumatologic manifestations of CHC (up to 38% of patients), and the differential diagnosis between the former and primary rheumatic diseases that may coexist in these patients [17].

In conclusion, we want to thank all of the authors that contributed to the present Special Issue, providing valuable articles on different virologic and clinical aspects relevant to the management of patients with CHC. 

## Data Availability

Not applicable.
